# Mental Health Help-Seeking and Associated Factors Among Public Health Workers During the COVID-19 Outbreak in China

**DOI:** 10.3389/fpubh.2021.622677

**Published:** 2021-05-11

**Authors:** Rui She, Xiaohui Wang, Zhoubin Zhang, Jinghua Li, Jingdong Xu, Hua You, Yan Li, Yuan Liang, Shan Li, Lina Ma, Xinran Wang, Xiuyuan Chen, Peien Zhou, Joseph Lau, Yuantao Hao, Huan Zhou, Jing Gu

**Affiliations:** ^1^Centre for Health Behaviours Research, Jockey Club School of Public Health and Primary Care, The Chinese University of Hong Kong, Hong Kong, China; ^2^School of Public Health, Lanzhou University, Lanzhou, China; ^3^Guangzhou Center for Disease Control and Prevention, Guangzhou, China; ^4^School of Public Health, Sun Yat-sen University, Guangzhou, China; ^5^Sun Yat-sen University Global Health Institute, School of Public Health and Institute of State Governance, Sun Yat-sen University, Guangzhou, China; ^6^Hubei Province Center for Disease Control and Prevention, Wuhan, China; ^7^School of Public Health, Nanjing Medical University, Nanjing, China; ^8^School of Public Health, Huazhong University of Science and Technology, Wuhan, China; ^9^Zigong Center for Disease Control and Prevention, Zigong, China; ^10^West China School of Public Health and West China Fourth Hospital, Sichuan University, Chengdu, China

**Keywords:** China, help-seeking, mental health, public health workers, COVID-19

## Abstract

**Background:** The COVID-19 outbreak in China has created multiple stressors that threaten individuals' mental health, especially among public health workers (PHW) who are devoted to COVID-19 control and prevention work. This study aimed to investigate the prevalence of mental help-seeking and associated factors among PHW using Andersen's Behavioral Model of Health Services Use (BMHSU).

**Methods:** A cross-sectional survey was conducted among 9,475 PHW in five provinces across China between February 18 and March 1, 2020. The subsample data of those who reported probable mental health problems were analyzed for this report (*n* = 3,417). Logistic and hierarchical regression analyses were conducted to examine the associations of predisposing, enabling, need, and COVID-19 contextual factors with mental health help-seeking.

**Results:** Only 12.7% of PHW reported professional mental help-seeking during the COVID-19 outbreak. PHW who were older, had more days of overnight work, received psychological training, perceived a higher level of support from the society, had depression and anxiety were more likely to report mental help-seeking (OR_m_ range: 1.02–1.73, all *p* < 0.05) while those worked in Centers for Disease Control and Prevention were less likely to seek help (OR_m_ = 0.57, *p* < 0.01). The belief that mental health issues were not the priority (64.4%), lack of time (56.4%), and shortage of psychologists (32.7%) were the most frequently endorsed reasons for not seeking help.

**Conclusions:** The application of BMHSU confirmed associations between some factors and PHW's mental health help-seeking. Effective interventions are warranted to promote mental health help-seeking of PHW to ameliorate the negative impact of mental illness and facilitate personal recovery and routine work.

## Introduction

The novel coronavirus disease (COVID-19) was declared a pandemic by the World Health Organization on March 12th, 2020 (1). To minimize transmissions through interpersonal contacts, the Chinese government has taken a series of severe control measures, including public transportation regulations, early detection of cases, contact tracing, and strict quarantine (2). These aggressive public health interventions contributed enormously to containing the epidemic and involve the efforts of public health workers (PHW) across the country. Since February 24th, 2020, the intensity of the COVID-19 epidemic in China has been greatly decreased and the daily new cases in China have been steadily declining (3). However, the ongoing community outbreak of COVID-19 in many other countries warrant continuous endeavors and research to tackle this global crisis.

While the COVID-19 outbreak affects many segments of the society (e.g., medical system, tourism, entertainment services, and educational institutions), it may result in strong mental distress, especially among healthcare workers. The shortage of medical resources, overwhelmed workload, high risk of infections, and fatigue can easily lead to severe mental health problems among front-line healthcare workers and PHW (4, 5). Previous studies have reported a high prevalence of depression (74%), anxiety (77%) (6), post-traumatic stress disorder (10%) (5), and sleeping problems (52%) (6) among healthcare workers during the outbreak of SARS (7, 8) when healthcare workers accounted for a fifth of all cases globally (9). During the COVID-19 outbreak period, a recent survey conducted in the epicenter Wuhan, China found that approximately 30% of front-line medical workers reported severe anxiety compared to general medical staff in another city (16%), where the epidemic was not serious (10). Another survey of PHW across five provinces in China also reported a high prevalence of depression (21.3%) and anxiety (19.0%) (11).

Despite the high mental distress, the culture of self-treatment, perception of stigma, lack of time, and fear of its consequences may delay appropriate help-seeking of healthcare workers and PWH (12, 13). Furthermore, PHW in China have undertaken a range of COVID-19 control and prevention tasks which might predispose them at high risk of infection and mental distress, including specimen collection, epidemiological investigation of patients, and isolation from their families. However, less attention and resources were allocated to PHW relative to frontline healthcare workers. To our best knowledge, no study is available to investigate the mental health help-seeking and associated factors among PHW who are at risk of mental health problems during the COVID-19 outbreak. Such information is implicative for effective interventions of help-seeking promotion targeted at PHW to ameliorate the negative impact of mental illness and allow for personal recovery and routine work (14–16).

To understand factors influencing an individual's health service utilization, the Andersen's Behavioral Model of Health Services Use (BMHSU) is one of the most widely acknowledged theoretical models. In general, health service utilization is a sequential and conditional function of three sets of factors: predisposing [e.g., age (17), sex (18) and education (19)], enabling [e.g., employment (20) and social support (21)], and need [e.g., self-perceived health (22) and chronic conditions (23)] factors. Predisposing factors reflect the individuals' propensity to utilize health services; enabling factors are the resources that may facilitate access to healthcare; and need factors represent potential needs of health service use. The BMHSU has frequently been used in health studies and applied in the research of mental health service utilization (23). In addition, during a critical phase of combating COVID-19, contextual factors related to the control and prevention work of COVID-19 such as workload and work-related risk and distress might play significant roles in PHW's mental health and help-seeking.

Thus, in the present study, we examined the prevalence and associated factors of mental health help-seeking using the framework of BMHSU among PHW who reported mental health problems, including (1) predisposing factors (e.g., age, sex, and work unit); (2) COVID-19 control and prevention work-related contextual factors (e.g., workload and work-related distress); (3) enabling factors (e.g., support and employment); (4) need factors (e.g., self-rated health and depression). Also, some perceived barriers against mental health help-seeking were investigated. It is hypothesized that PHW would have a low prevalence of mental health help-seeking and report multiple barriers to professional mental health services. Predisposing, COVID-19, enabling, and need-related factors would be significantly associated with help-seeking behavior.

## Materials and Methods

### Study Design and Data Collection

A cross-sectional survey was conducted from February 18 to March 1, 2020, among PHW during the COVID-19 epidemic phase. As reported previously, to increase the representativeness of PHW in China, data were collected from five provinces (Hubei, Guangdong, Sichuan, Jiangsu, and Gansu) which were purposely selected to cover different levels of epidemic severity defined by numbers of reported cases and to cover different regions of the country (center, southern, western, eastern, and northern). Three to five cities were conveniently selected per province; within the selected cities, three to five districts/counties and five to 10 subdistricts/towns were further selected to represent both different outbreak levels and different regions. Inclusion criteria were: (1) aged 18 years old or above; (2) working at the public health system [i.e., centers for Disease Control and Prevention (CDC) or primary healthcare institutes] of the selected places during the study period; (3) participated in COVID-19 control and prevention work. The online survey link was distributed by site investigators (e.g., CDC staff) through WeChat/QQ working groups of PHW in each city, which are the most commonly used social media applications in China (24). All participants were informed about the study's background, anonymity, restriction to academic use, and that return of the completed questionnaire implied informed consent. Of all the 9,475 returned questionnaires, 3,417 (36.1%) participants reported probable mental health problems (responded “yes” or “unsure” to the question assessing perceived mental health concerns) and were thus included in the analysis. The study was approved by the ethics committee of the School of Public Health, Sun Yat-sen University (Reference No.: 2020-012).

### Measurements

#### Predisposing Characteristics (Independent Variables)

Socio-demographic information collected included age, sex, provinces, work units, and whether or not having children under 6 years old.

#### COVID-19 Control and Prevention Work-Related Factors (Independent Variables)

Information about their work in terms of work contents, training, workload, and work-related distress was collected, which were listed in detail below.

1) *Workload*. Participants were asked about the number of days of overnight work due to COVID-19 related work and average working hours per day during the past week.
2) *Work-related risk and distress*. (i) Involvement in fieldwork with risk exposure: A list of questions including 14 types of fieldwork were investigated. Those who checked the items including specimen collection or face-to-face epidemiological survey of patients were considered to have risk exposure; (ii) Worries about getting infected at work: Three items were used to assess the level of concerns of themselves being infected and family members being infected because of them, and family members' concerns about them being infected. Responses were rated on a 5-point Likert scale (1 = almost none to 5 = extremely high); (iii) Work-related distress: Five self-constructed items were used to measure perceived pressure, which were rated on a 5-point Likert scale (1 = almost none to 5 = extremely high). Example items include “Your work is not being understood sometimes” and “You feel being treated unfairly in the workplace.” Cronbach's alpha was 0.81 in the present study.
3) *Psychological training*. Participants were asked whether they had received any psychological training about how to cope with the COVID-19 outbreak.
4) *Personal attitudes toward work*. Two items were used to assess whether the PHW agreed that involvement in COVID-19 control and prevention work would facilitate their future personal development and would be a manifestation of their personal ability. Responses were rated on a 5-point Likert scale (1 = strongly disagree to 5 = strongly agree) and dichotomized into low (1–3 point) and high level of consent (4–5 point).

#### Enabling Factors (Independent Variables)

1) *Support*. Three items were used to access the support participants perceived from the workplace, including technical support, logistic support, and mental support during the COVID-19 outbreak period. Another three questions were asked to access perceived support from their colleagues, families, and society. Responses were rated on a 5-point Likert scale (1 = none to 5 = very high) and recoded into low (1–3 points) and high level of support (4–5 points).
2) *Employment*. The job title was categorized as junior, intermediate, senior/deputy senior, and others (e.g., volunteers or temporary workers).

#### Need Factors (Independent Variables)

1) *Self-rated health status*. A single item was used to measure the self-perceived health condition during the last week, with a 5-point response scale (very good, good, fair, poor, very poor). Similar measurement has been widely used in China and other countries (25, 26).
2) *Physical fatigue*. A single item was used to assess the level of physical fatigue, with a 5-point Likert scale (not at all, a few, medium, high, very high).
3) *Probable depression*. The validated Chinese version of the Patient Health Questionnaire (PHQ-9) was used to assess the level of depression. Participants were asked to report how frequently they had experienced nine core depressive symptoms in the past 2 weeks (11). Items are assessed on 4-point Likert-type scales, ranged from not at all (0) to nearly every day (3). A cut-off summative score of ≥10 was used to define the presence of moderate-severe depressive symptoms (27). The Cronbach's α in this study was 0.92.
4) *Probable anxiety*. The Generalized Anxiety Disorder 7-item scale (GAD-7) was used to evaluate symptoms of generalized anxiety, which has been validated and applied in previous studies in China (28, 29). Symptom frequency was reported on a 4-point Likert-type scale ranging from never (0) to nearly every day (3). A score of 10 is taken as the cut-off point for the presence of probable moderate-severe anxiety (30). The Cronbach's α was 0.93 in this study.

#### Mental Health Help-Seeking (Dependent Variable)

Participants were asked whether they had managed to seek help from mental health professionals during the COVID-19 outbreak phase. The outcome was coded as a binary variable: those who sought help from professionals and those who did not (0 = no and 1 = yes).

#### Perceived Barriers Against Mental Health Help-Seeking

Of the respondents who had not sought help from mental health professionals, they were further asked the potential reasons. Five items on perceived barriers were presented, including lack of time, shortage of mental health professionals, feeling that the treatment is useless, belief of that psychological problems were not the main issues at the moment, and not knowing how to access mental health care.

The complete text of measurement is available in Supplementary Table 1 and the original questionnaire in Chinese will be made available upon request from the corresponding author.

### Statistical Analysis

All the statistical analyses were performed using SPSS 25.0 Statistics for Windows (IBM Corp. Released 2018, Armonk, NY: IBM Corp), with two-tailed *p* values < 0.05 being considered to be statistically significant. Descriptive data of participants' characteristics were firstly presented, including sociodemographic, COVID-19 related work, support, health status, and mental health help-seeking. Logistic regression was used to test the factors associated with mental health help-seeking, including predisposing, COVID-19 work, enabling, and need factors. Univariate odds ratio (ORu), adjusted odds ratio (AOR) controlling for all predisposing variables, and corresponding 95% confidence intervals (CI) were reported.

In addition, hierarchical multiple logistic regression analysis was conducted to test the incremental variance of mental health help-seeking behavior by a set of independent variables. In a four-block model, predisposing factors (e.g., province, sex, and age) were entered into the first block, followed by COVID-19 control and prevention work-related factors (e.g., work content and work-related distress) entered in block 2, enabling factors (e.g., perceived support and employment) entered in block 3, and need factors (e.g., self-rated health status and depression) entered in block 4. Change in the statistic −2^*^Log-Likelihood (−2LL) and chi-square (χ^2^) was used to test the significance of each block in explaining the dependent variable of help-seeking. Lastly, we described barriers to mental health help-seeking help among those who had not sought professional mental health support.

## Results

### Characteristics of the Study Participants

Of the 3,417 participants who reported mental health concerns, 66.2% were female; the mean age was 39.1 [standard deviation (SD) 9.5]; 27.7% had children under 6 years old. The majority of participants (61.9%) were staff in primary healthcare institutes, 35.4% worked in disease control and health education departments (CDC staff), and 44.7% had a junior employment title. About half (46.1%) had engaged in fieldwork with infection risk during the COVID-19 outbreak. The mean working hour in the past week was 10.0 h (SD 2.9) and participants had worked overnight for 2.9 times averagely (SD 2.7). About half of PHW had received psychological training about how to cope with COVID-19, and the majority believed that the engagement in COVID-19 control and prevention work was beneficial to their future development (53.1%) and a manifestation of personal ability (74.8%).

About one-fifth perceived a poor/very poor health status and 40.7% reported a high level of physical fatigue. The prevalence of probable depression and anxiety was 40.6 and 37.3%, respectively. The majority (53.2–64.4%) perceived a low level of support from the workplace, colleagues, society, and 73.4% perceived a high level of support from families. Only 12.7% of the participants reported professional mental health help-seeking (Table 1).

**Table 1:** Characteristics of health care workers who perceived probable mental health problems (*N* = 3,417).

	***N* (%)/*M* ±*SD***
**Predisposing factors**	
Province	
Hubei	558 (16.3)
Guangdong	1,071 (31.3)
Jiangsu	354 (10.4)
Sichuan	1,144 (33.5)
Gansu	290 (8.5)
Sex	
Male	1,155 (33.8)
Female	2,262 (66.2)
Age (years)	39.1 ± 9.5
Having children under 6 years (yes)	946 (27.7)
Work unit	
Primary care	2,116 (61.9)
Disease control and health education	1,209 (35.4)
Health administration	38 (1.1)
Others	54 (1.6)
**COVID-19 prevention and control work-related factors**	
Workload	
Days of overnight work due to COVID-19 outbreak	2.9 ± 2.7
Average working hours per day in the past week	10.0 ± 2.9
Work-related risk and distress	
Involvement in filed work with risk exposure	
No	1,842 (53.9)
Yes	1,575 (46.1)
Worries about getting infected at work	7.4 ± 2.7
Work-related distress	8.9 ± 3.6
Training of psychological coping with COVID-19 outbreak	
No	1,994 (58.4)
Yes	1,423 (41.6)
Personal attitudes toward work	
Consent of personal future development	
Low level	1,603 (46.9)
High level	1,814 (53.1)
Consent of manifestation of the ability	
Low level	861 (25.2)
High level	2,556 (74.8)
**Enabling factors**	
Perceived support from the workplace	
Low level	1,817 (53.2)
High level	1,600 (46.8)
Perceived support from colleagues	
Low level	1,833 (53.6)
High level	1,584 (46.4)
Perceived support from families	
Low level	910 (26.6)
High level	2,507 (73.4)
Perceived support from society	
Low level	2,200 (64.4)
High level	1,217 (35.6)
Employment title	
Junior	1,529 (44.7)
Intermediate	1,078 (31.5)
Senior/Deputy senior	412 (12.1)
Others (volunteer etc.)	398 (11.6)
**Need factors**	
Self-rated health status during the last week	
Very good/good	1,270 (37.1)
Fair	1,519 (44.5)
Very poor/Poor	628 (18.4)
Level of physical fatigue	
Not at all/A few/Medium	2,027 (59.3)
High/Very High	1,390 (40.7)
Probable anxiety	
No	1,660 (62.7)
Yes	988 (37.3)
Probable depression	
No	1,572 (59.4)
Yes	1,076 (40.6)
Mental health help-seeking	
No	2,984 (87.3)
Yes	433 (12.7)

*Data are n (%), unless otherwise specified*.

### Univariate and Adjusted Analysis of Factors Associated With Mental Health Help-Seeking

1) Predisposing factors: Of all the socio-demographic factors, only the work unit of CDC was negatively associated with mental health help-seeking (ORu = 0.69, 95% CI 0.55–0.87, reference group: primary healthcare institution) in univariate analysis.
2) COVID-19 control and prevention work-related factors: As shown in Table 2, six COVID-19 work-related factors including days of overnight work, work-related distress, worries about getting infected at work, psychological training, two variables related to positive attitudes toward COVID-19 work were consistently and significantly associated with a higher likelihood of mental health help-seeking in the bivariate (ORu range: 1.07–1.41, all *p* < 0.01) and adjusted logistic regression analysis controlling for all predisposing variables (AOR range: 1.06–1.40, all *p* < 0.05).
3) Enabling factors: Only perceive support from society was positively associated with mental health help-seeking with marginal significance in univariate analysis (ORu = 1.21, 0.05 < *p* < 0.10) but the association was not significant in the adjusted analysis.
4) Need factors: All need indicators including poor self-perceived health status, high level of physical fatigue, and the presence of depression and anxiety showed significant and positive associations with mental health help-seeking in the bivariate analysis (ORu range: 1.03–2.23, all *p* < 0.01). The significance of these associations remained in adjusted analysis (AOR range: 1.07–2.35, all *p* < 0.05) (Table 2).

**Table 2:** Univariate and multivariate analysis of factors associated with mental health help-seeking among public health workers who reported probable mental health problems (*N* = 3,417).

**Independent variables**	**ORu (95% CI)**	**AOR (95% CI)**
**Predisposing factors**		
Province		
Hubei	1.00 (reference)	NA
Guangdong	0.78 (0.57, 1.07)	
Jiangsu	1.32 (0.91, 1.92)	
Sichuan	1.05 (0.78, 1.42)	
Gansu	1.02 (0.67, 1.55)	
Sex		
Male	1.00 (reference)	NA
Female	0.85 (0.69, 1.05)	
Age (year)	1.01 (1.00, 1.02)^†^	NA
Having children under 6 years (yes)		
No	1.00 (reference)	NA
Yes	0.91 (0.73, 1.15)	
Work unit		
Primary care	1.00 (reference)	NA
Disease control and health education	0.69 (0.55, 0.87)^**^	
Health administration	1.65 (0.75, 3.63)	
Other	1.07 (0.50, 2.30)	
**COVID-19 prevention and control work-related factors**		
Workload		
Days of overnight work due to COVID-19 outbreak	1.07 (1.03, 1.10)^***^	1.06 (1.02, 1.10)^**^
Average Working hours per day in the past week	1.04 (1.00, 1.07)^*^	1.04 (1.00, 1.07)^†^
Work-related risk and distress		
Involvement in filed work with risk exposure		
No	1.00 (reference)	1.00 (reference)
Yes	1.09 (0.89, 1.34)	1.09 (0.88, 1.33)
Worries about getting infected at work	1.07 (1.03, 1.12)^***^	1.07 (1.03, 1.11)^**^
Work related distress	1.07 (1.04, 1.10)^***^	1.07 (1.04, 1.10)^***^
Training of psychological coping with COVID-19 outbreak		
No	1.00 (reference)	1.00 (reference)
Yes	1.41 (1.20, 1.66)^***^	1.32 (1.12, 1.55)^**^
Personal attitudes toward work		
Consent of personal future development		
Low level	1.00 (reference)	1.00 (reference)
High level	1.35 (1.10, 1.66)^**^	1.40 (1.14, 1.72)^**^
Consent of manifestation of the ability		
Low level	1.00 (reference)	1.00 (reference)
High level	1.39 (1.09, 1.79)^**^	1.39 (1.08, 1.79)^*^
**Enabling factors**		
Perceived support from the workplace		
Low level	1.00 (reference)	1.00 (reference)
High level	0.86 (0.71, 1.06)	0.89 (0.72, 1.09)
Perceived support from colleagues		
Low level	1.00 (reference)	1.00 (reference)
High level	0.85 (0.69, 1.04)	0.86 (0.70, 1.06)
Perceived support from families		
Low level	1.00 (reference)	1.00 (reference)
High level	0.89 (0.71, 1.12)	0.90 (0.71, 1.12)
Perceived support from society		
Low level	1.00 (reference)	1.00 (reference)
High level	1.21 (0.98, 1.49)^†^	1.18 (0.95, 1.45)
Employment title		
Junior	1.00 (reference)	1.00 (reference)
Intermediate	0.85 (0.67, 1.07)	0.91 (0.71, 1.18)
Senior/Deputy senior	0.89 (0.64, 1.24)	0.97 (0.67, 1.41)
Others (volunteer etc.)	0.99 (0.72, 1.37)	0.99 (0.71, 1.38)
**Need factors**		
Self-rated health status during the last week		
Very good/good	1.00 (reference)	1.00 (reference)
Fair	1.03 (0.82, 1.30)	1.07 (0.85, 1.35)
Very poor/Poor	1.49 (1.13, 1.95)^**^	1.57 (1.19, 2.09)^**^
Level of physical fatigue		
Not at all/A few/Medium	1.00 (reference)	1.00 (reference)
High/Very high	1.37 (1.12, 1.67)^**^	1.41 (1.15, 1.74)^**^
Probable anxiety		
No	1.00 (reference)	1.00 (reference)
Yes	2.23 (1.78, 2.79)^***^	2.35 (1.87, 2.96)^***^
Probable depression		
No	1.00 (reference)	1.00 (reference)
Yes	1.91 (1.53, 2.40)^***^	2.24 (1.77, 2.83)^***^

† *0.05< p <0.01*,

* *p < 0.05*,

** *p < 0.01*,

*** *p < 0.001, NA: Not applicable*.

*ORu, Univariate Odds Ratio; AOR, Odds ratio adjusted for predisposing variables*.

### Hierarchical Regression Analysis

Table 3 presents the final results of the hierarchical multiple logistic regression models for mental health help-seeking. Except for Block 3 (χ^2^ = 11.66, *p* = 0.112), surplus blocks of the independent variables significantly contributed to the variance of the help-seeking outcome (range for block χ^2^: 22.68–43.14, all *p* < 0.01). The final hierarchical multiple regression model showed that of the twenty-two independent variables (Model 4), seven variables were significantly associated with mental health help-seeking: (1) age (ORm = 1.02, 95% CI = 1.01–1.03), (2) working in disease control and health education department (ORm = 0.57, 95% CI = 0.43–0.76), (3) days of overnight work (ORm = 1.05, 95% CI = 1.00–1.09), (4) getting training of psychological coping with COVID-19 outbreak (ORm = 1.22, 95% CI = 1.03–1.45), (5) perceiving support from society (ORm = 1.35, 95% CI = 1.01–1.80), (6) the presence of probable depression (ORm = 1.49, 95% CI = 1.10–2.02), and (7) probable anxiety (ORm = 1.73, 95% CI = 1.28–2.33).

**Table 3:** Results of hierarchical regression analyses of factors associated with mental health help-seeking among public health workers who reported probable mental health problems (*N* = 3,417).

**Independent variables**	**Professional mental health help-seeking**			
	**Model 1**	**Model 2**	**Model 3**	**Model 4**
**Block 1 Predisposing characteristics**				
Province (Hubei vs. others)	1.13 (0.85, 1.49)	1.09 (0.82, 1.45)	1.07 (0.81, 1.43)	1.13 (0.84, 1.52)
Sex (Male vs. female)	0.84 (0.66, 1.07)	0.90 (0.70, 1.16)	0.90 (0.70, 1.17)	0.91 (0.70, 1.18)
Age (years)	1.01 (1.00, 1.03)*	1.02 (1.00, 1.03)*	1.01 (1.00, 1.03)*	1.02 (1.01, 1.03)**
Having children under 6 years (No vs. yes)	1.03 (0.77, 1.36)	0.98 (0.74, 1.30)	0.99 (0.74, 1.33)	0.97 (0.73, 1.30)
Work unit				
Primary care	1.00 (reference)	1.00 (reference)	1.00 (reference)	1.00 (reference)
Disease control and health education	0.65 (0.51, 0.83)^**^	0.68 (0.52, 0.89)^*^	0.70 (0.52, 0.92)^*^	0.57(0.43, 0.76)^***^
Health administration	1.53 (0.61, 3.82)	1.38 (0.54, 3.54)	1.43 (0.55, 3.68)	1.44 (0.56, 3.72)
Others	0.83 (0.32, 2.14)	0.88 (0.34, 2.31)	0.85 (0.32, 2.23)	0.87 (0.33, 2.31)
**Block 2 COVID-19 control and prevention work-related factors**				
Days of overnight work due to COVID-19 outbreak		1.05 (1.01, 1.10)^*^	1.05 (1.01, 1.10)	1.05 (1.00, 1.09)^*^
Average Working hours per day in the past week		1.00 (0.96, 1.05)	1.00 (0.96, 1.05)	1.00 (0.96, 1.05)
Involvement in filed work with risk exposure (No vs. yes)		0.93 (0.74, 1.18)	0.95 (0.75, 1.20)	0.95 (0.75, 1.20)
Worries about getting infected at work		1.03 (0.99, 1.08)	1.04 (0.99, 1.09)	1.01 (0.96, 1.06)
Work-related distress		1.06 (1.02, 1.09)^**^	1.05 (1.02, 1.09)^**^	1.02 (0.98, 1.05)
Training of psychological coping with COVID-19 outbreak (No vs. yes)		1.29 (1.08, 1.55)^**^	1.30 (1.08, 1.56)^**^	1.22 (1.03, 1.45)^*^
Consent of personal future development (Low vs. high)		1.23 (0.95, 1.59)	1.24 (0.96, 1.62)	1.53 (0.96, 1.97)
Consent of manifestation of ability (Low vs. high)		1.12 (0.82, 1.54)	1.16 (0.84, 1.60)	0.77 (0.54, 1.09)
**Block 3 Enabling factors**				
Perceived support from the workplace (Low vs. high)			0.88 (0.65, 1.18)	0.92 (0.68, 1.25)
Perceived support from colleagues (Low vs. high)			0.76 (0.56, 1.04)	0.74 (0.54, 1.02)
Perceived support from families (Low vs. high)			0.83 (0.61, 1.13)	0.87 (0.64, 1.19)
Perceived support from society (Low vs. high)			1.37 (1.02, 1.82)^*^	1.35 (1.01, 1.80)^*^
Employment title				
Junior			1.00 (reference)	1.00 (reference)
Intermediate			0.91 (0.68, 1.23)	0.88 (0.65, 1.19)
Senior/Deputy senior			1.17 (0.77, 1.76)	1.21 (0.80, 1.83)
Others (volunteer etc.)			1.01 (0.70, 1.45)	1.01 (0.70, 1.46)
**Block 4 Need factors**				
Self-rated health status during the last week				
Very good/good				1.00 (reference)
Fair				1.01 (0.70, 1.46)
Very poor/Poor				1.20 (0.84, 1.71)
Physical fatigue (Low vs. high)				1.00 (0.76, 1.31)
Probable anxiety (No vs. yes)				1.73 (1.28, 2.33)^***^
Probable depression (No vs. yes)				1.49 (1.10, 2.02)^**^
**Chi-square change**	22.68	43.14	11.66	43.07
**Block significance**	0.002	<0.001	0.112	<0.001
**−2 Log likelihood**	2,067.88	2,024.73	2,013.08	1,970.01

* *p < 0.05*,

** *p < 0.01*,

*** *p < 0.0.01*.

### Mentioned Barriers to Mental Health Help-Seeking

Of the 2,984 (87.3%) participants who had not sought help from mental health professionals, the belief that mental health issues were not the priority at the moment was the most often endorsed reason (64.4%), followed by lacking time (56.4%), shortage of mental health professionals (32.7%), lack of access to mental health services (17.5%), and feeling of that treatment was useless (12.7%).

## Discussion

To our knowledge, the present study was the first one to examine the mental health help-seeking and associated factors in a large sample of PHW who perceived probable mental health problems during the COVID-19 outbreak in China. The prevalence of mental health help-seeking was alarmingly low (12.7%), compared to a prevalence of 48% among Chinese adults with mental disorders reported in a national survey and 84% of clinical psychologists who experienced mental problems in the UK (21). Both service provision and health promotion are greatly warranted. Predisposing (e.g., age and work unit), enabling (e.g., perceived support from society), need (e.g., depression), and COVID-19 work-related contextual factors (e.g., overnight work and psychological training) were significantly associated with mental health help-seeking. When the four dimensions of factors were examined with a series of hierarchical regression models, the variables overall maintained their level of significance with the inclusion of subsequent blocks of variables, indicating substantial independence across underlying constructs. The findings provided empirical support for the application of BMHSU among PHW and implications for intervention to promote help-seeking behaviors of PHW.

Among predisposing factors, increased age was associated with increased mental help-seeking, consistent with prior literature (31–33). Participants who were CDC workers were less likely to seek help from mental health professionals compared to those working in primary healthcare institutions, as they might bear more responsibilities in regional disease prevention. This argument is supported by the findings that a larger proportion of CDC workers endorsed lack of time and mental health problems not being the priority issues as the reasons for not seeking professional mental health help relative to PHW in primary care institutions (data not shown). These associations remained significant in the final hierarchical regression model, suggesting that attention should be especially paid to younger CDC workers with mental health service needs to promote their help-seeking behaviors.

The COVID-19 control and prevention workload was high, evidenced by an averaged 10 working hours per day, 3 times of overnight work, and high levels of distress and infection concerns. These COVID-19 work-related stressors were associated with a higher likelihood of mental health help-seeking in the adjusted analysis, which is consistent with previous studies that stress may indicate higher mental health needs and prompt help-seeking behaviors (34–37). In addition, about 40% of PHW had received training about psychological coping with COVID-19 and over half held positive attitudes toward their COVID-19 work; such positive attitudes and psychological training were positively associated with help-seeking in the adjusted analysis. In the final hierarchical model, of all the COVID-19 work contextual factors, only days of overnight work and psychological training remained as a significant factor of help-seeking. It is plausible that the associations between positive attitudes toward COVID-19 work and help-seeking in the adjusted analysis might be attributed to the confounding effect of psychological training as those who received psychological training might be more likely to have positive reframing about their work and situations. Corroborating previous studies (16, 21, 38, 39), the findings demonstrated the independent effect of psychological training on help-seeking, which implies that providing essential training is a feasible and effective approach to promote help-seeking of PHW.

Consistent with previous studies (40, 41), social support from society was positively associated with mental health help-seeking. Studies have shown that social support, being prompted to seek help, and knowing someone who has sought help are associated with positive attitudes toward seeking mental health services (42, 43) as well as perceived need and the actual behavior of mental health help-seeking (23, 43, 44). Thus, social support may have increased the flow of information and advice and established subjective norms related to mental health service utilization (45). Notably, of the various sources of social support investigated, perceived support from the society was relatively low but was the only significant factor associated with help-seeking. Therefore, to increase the general public's understanding and appreciation of the efforts of PHW is especially important for both epidemic control and the well-being of PHW. Transparent and thoughtful communication with the community might contribute to trust and reduce negative emotions toward PHW when implementing some COVID-19 control work such as isolation of close contacts and home inspections. Support and encouragement from the public (e.g., via social media) for the PHW could reduce their mental distress and facilitate help-seeking behaviors. In contrast, the employment title did not show association with help-seeking, indicating that employment may not be a barrier to mental health care.

We found relatively poor evaluated health among PHW who reported probable mental health concerns, with a prevalence of 40% of anxiety and depression among participants as well as a high level of physical fatigue. Corroborating prior literature (46, 47), need factors were strong predictors of health service utilization. These physical and mental health indicators showed consistent and significant associations with mental health help-seeking in the adjusted analysis. In addition, depression and anxiety remained significant in the final hierarchical regression model with all potential variables included. The findings are congruent with previous studies that psychological distress and the presence of depression could increase the likelihood of seeking professional help (48, 49). Moreover, the hierarchical regression models showed that the COVID-19 work-related distress turned into non-significance when including need factors (e.g., depression), suggesting that COVID-19 work stress might have an indirect effect on mental health help-seeking via depression and anxiety.

The participants endorsed a range of barriers against mental health help-seeking, including the belief that mental illness was not the priority at the moment and lack of time. These might be attributed to the great workload of PHW involved in the COVID-19 control and prevention work, which resulted in the ignorance of their own health needs. Other barriers such as shortage of mental health professionals and feeling that treatment was useless were identified, similar to previous studies that reported the lack of access to psychiatrists and related facilities in China (50). Mental illness is often conceived as a representation of personal weakness or lack of willpower in Chinese culture, hence discourages disclosure and help-seeking related to mental health problems (51). Such barriers to mental health treatment persisted during the pandemic and intertwined with work strain and restrictions due to COVID-19 (e.g., quarantine) (52). Tailored interventions including linkage to mental health care and training to alter misconceptions about mental disorders are essential to overcome these barriers.

The present study has several limitations. First, the design of the cross-sectional study did not enable the interpretation of causality. Longitudinal studies are warranted to examine the predictors of service utilization. Second, although we recruited a large sample of public health workers across five provinces, the results might not apply to the general PHW with a non-random sampling framework. In addition, selection bias may exist as those experiencing work strain may be less likely to engage with their WeChat group and underrepresented in this study. Third, self-reported information might have induced misclassification due to recall bias and social desirability. Fourth, single-item measures were used to screen PHW with perceived mental health concerns instead of confirmed diagnoses and to assess help-seeking behavior. Future studies with validated questionnaires to measure these constructs are recommended.

In summary, this study revealed a low prevalence of mental health help-seeking among PHW who reported probable mental health concerns, and confirmed associations between some predisposing characteristics, enabling resources, need, and COVID-19 contextual factors on PHW's help-seeking. Healthcare professionals experience high levels of work stress in their regular work but most of them would be reluctant to disclose mental health issues or seek professional help for a number of reasons. A similar case occurred to PHW during the COVID-19 pandemic. Nevertheless, the reasons and factors for the low level of mental health services utilization among healthcare professionals and PHW have been scarcely explored. The present paper adds valuable data to the field of healthcare services utilization of health professionals during high-stress situations and identifies potential focus areas for future interventions.

## Data Availability Statement

The raw data supporting the conclusions of this article will be made available by the authors, without undue reservation.

## Ethics Statement

The study was approved by the ethics committee of the School of Public Health, Sun Yat-sen University (Reference No.: 2020-012). Informed consents were obtained online before self-administering the questionnaire by clicking a survey link after being briefed about the background, aims, anonymous nature and length of the survey.

## Author Contributions

Resources and data were obtained by XHW, ZZ, JX, HY, YaL, YuL, SL, LM, HZ, and JG. Statistical analysis was performed by RS, XRW, XC, and PZ. The first draft of the manuscript was written by RS, XRW, XC, and PZ, and all authors commented on previous versions of the manuscript. All authors contributed to the study conception and design, read and approved the final manuscript.

## Conflict of Interest

The authors declare that the research was conducted in the absence of any commercial or financial relationships that could be construed as a potential conflict of interest.
