# Supplementary material for: Mental Health Help-Seeking and Associated Factors Among Public Health Workers During the COVID-19 Outbreak in China
Source: Front Public Health. 2021 May 11;9:622677. doi: 10.3389/fpubh.2021.622677 (PMC8144452; doi:10.3389/fpubh.2021.622677)
Supplement: Supplementary file 1 [file Table_1.DOCX]

Supplementary Table 1a Measurements of the study variables

| Variable label | Survey question | Responses |
| --- | --- | --- |
| Overnight work | How many days have you been working all night due to the COVID-19-related work? | 0, 1, 2, 3, 4, 5, 6, 7, >7 |
| Working hour | In the past week, how many hours have you been working every day on average? | <=4, 5, 6, 7, 8, 9, 10, 11, 12, 13, 14, 15, 16, 17, 18, 19, >=20 |
| Involvement in fieldwork | What types of fieldwork related to COVID-19 control and prevention have you participated in? (please check all items that apply to you) | face-to-face epidemiological investigations of the patients and close contacts, performance of epidemiological investigations by telephone or video calls, medical observation of the close contacts, collection and shipment of specimens, provision of health education, and performance of community-based investigations. |
| Worries about getting infected at work | (1) To what extent have you been worrying about being infected with COVID-19 during work?  (2) To what extent have you been worrying about family members being infected with COVID-19 via you?  (3) To what extent have your family members been worrying about you being infected with COVID-19? | Almost none, few, medium, high, extremely high |
| Work-related distress | During the COVID-19 control and prevention work, to what extent do you have any below-mentioned concerns?  (1) Your work is not being understood sometimes.  (2) You feel being treated unfairly in the workplace.  (3) You feel wronged sometimes in the workplace.  (4) Family members can’t understand your contribution.  (5) You have concerns about other regular work in addition to COVID-19-related work. | Almost none, few, medium, high, extremely high |
| Psychological training | Have you received any training about psychological coping and response to the COVID-19 pandemic? | No, yes |
| Personal attitudes towards work | (1) Involvement in COVID-19 control and prevention work would facilitate your future personal development.  (2) Involvement in COVID-19 control and prevention work would be a manifestation of your personal ability. | Strongly disagree, disagree, neutral, agree, strongly agree |
| Support | During the COVID-19 control and prevention work, how much support have you received?  (1) Technical support from the workplace  (2) Logistic support from the workplace  (3) Mental support from the workplace  (4) Support from colleagues  (5) Support from family members  (6) Support from society | None, few, medium, high, extremely high |
| Employment | What is your job seniority rank? | Junior, intermediate, senior, deputy senior, and others (e.g., volunteers or temporary workers) |
| Self-rated health status | How is your health in general in the past week? | Very good, good, fair, poor, very poor |
| Physical fatigue | To what extent have you felt physical fatigue? | Almost none, few, medium, high, extremely high |
| Mental health help-seeking | Have you ever managed to seek help from mental health professionals during the COVID-19 outbreak phase? | No, yes |
| Perceived barriers against mental health help-seeking | What are the reasons for not seeking help from mental health professionals? (please check all items that apply to you) | lack of time, shortage of mental health professionals, feeling that the treatment is useless, belief of that psychological problems were not the main issues at the moment, and not knowing how to access mental health care |
